# Model-guided therapy for hepatocellular carcinoma: a role for information technology in predictive, preventive and personalized medicine

**DOI:** 10.1186/1878-5085-5-16

**Published:** 2014-09-23

**Authors:** Leonard Berliner, Heinz U Lemke, Eric vanSonnenberg, Hani Ashamalla, Malcolm D Mattes, David Dosik, Hesham Hazin, Syed Shah, Smruti Mohanty, Sid Verma, Giuseppe Esposito, Irene Bargellini, Valentina Battaglia, Davide Caramella, Carlo Bartolozzi, Paul Morrison

**Affiliations:** 1 New York Methodist Hospital, Brooklyn, NY 11215, USA; 2 Weill Medical College of Cornell University, New York, NY 10021, USA; 3 Technical University of Berlin, 10623 Berlin, Germany; 4 University of Southern California, Los Angeles, CA, 90089, USA; 5 David Geffen School of Medicine at UCLA, Los Angeles, CA 90095, USA; 6 University of Arizona College of Medicine, Tucson, AZ, 85724, USA; 7 Georgetown University Medical Center, Washington, DC 20007, USA; 8 University Hospital Pisa, University of Pisa (I), 56126 Pisa, Italy; 9 Brigham and Women's Hospital, Boston, MA 02115, USA

**Keywords:** Personalized medicine, Hepatocellular carcinoma, Information technology, Model-guided therapy, Therapy imaging and model management system, TIMMS, Digital patient model, Patient-specific model, Model-based medical evidence, Bayesian network

## Abstract

Predictive, preventive and personalized medicine (PPPM) may have the potential to eventually improve the nature of health care delivery. However, the tools required for a practical and comprehensive form of PPPM that is capable of handling the vast amounts of medical information that is currently available are currently lacking. This article reviews a rationale and method for combining and integrating diagnostic and therapeutic management with information technology (IT), in a manner that supports patients through their continuum of care. It is imperative that any program devised to explore and develop personalized health care delivery must be firmly rooted in clinically confirmed and accepted principles and technologies. Therefore, a use case, relating to hepatocellular carcinoma (HCC), was developed. The approach to the management of medical information we have taken is based on model theory and seeks to implement a form of model-guided therapy (MGT) that can be used as a decision support system in the treatment of patients with HCC. The IT structures to be utilized in MGT include a therapy imaging and model management system (TIMMS) and a digital patient model (DPM). The system that we propose will utilize patient modeling techniques to generate valid DPMs (which factor in age, physiologic condition, disease and co-morbidities, genetics, biomarkers and responses to previous treatments). We may, then, be able to develop a statistically valid methodology, on an individual basis, to predict certain diseases or conditions, to predict certain treatment outcomes, to prevent certain diseases or complications and to develop treatment regimens that are personalized for that particular patient. An IT system for predictive, preventive and personalized medicine (ITS-PM) for HCC is presented to provide a comprehensive system to provide unified access to general medical and patient-specific information for medical researchers and health care providers from different disciplines including hepatologists, gastroenterologists, medical and surgical oncologists, liver transplant teams, interventional radiologists and radiation oncologists. The article concludes with a review providing an outlook and recommendations for the application of MGT to enhance the medical management of HCC through PPPM.

## Review

### Introduction

Predictive, preventive and personalized medicine (PPPM) may have the potential to eventually improve the nature of health care delivery. This article was initially conceived, and has gradually evolved, to seek solutions to issues and tasks relating to information technology (IT) and predictive, preventive and personalized medicine (PPPM) as identified in the White Paper 2012 of the European Association for Predictive, Preventive and Personalised Medicine (EPMA) [1]. Specifically, this article reviews work being done to define the requirements and interrelationships between PPPM, clinical medical practice and basic medical research that could be best served by information technology (IT) [2,3]. To avoid the problems inherent in formulating IT solutions in isolation, a use case was developed employing hepatocellular carcinoma (HCC). It may be useful to define how the term use case is employed here. The term ‘use case’ has been used in software and systems engineering to describe a system's behavior and appearance and to diagram the main flow of events in a system as it responds to stimulus [4,5]. A use case can be used both to define conceptual requirements of a system and to evaluate compliance with user requirements during testing and evaluation activities [4]. Software and systems engineering principles relating to use cases have been extended and applied to medical applications, for example, to establish a foundation for evaluating information design in electronic health record (EHR) applications [4]. In this article, a use case has been adapted to describe the functional and structural requirements of an information technology system for predictive, preventive and personalized Medicine (ITS-PM) by means of generating a digital patient model (DPM) from available medical data sources. The use case maintains a focus on the IT aspects of the system under development, by limiting the DPM to entities relating to HCC.

The subject matter of this article was approached from four separate, but interrelated, tasks: (1) to review current understanding and clinical practices relating to HCC; (2) to propose an IT system to deal with the vast amount of information relating to HCC, including clinical decision support and research needs; (3) to determine the ways in which a clinical liver cancer centre can contribute to this IT approach; and (4) to explore the enhancements and impact that the first three tasks, and therefore PPPM, may have on the management of HCC.

One of the goals of this article is to provide an overview of a roadmap that is under development for the generation of IT tools and methodologies to facilitate PPPM [3]. Our approach to the management of medical information is based on model theory that has arisen from a conceptual transformation from image-guided patient management to more complex model-guided patient management. This approach seeks to implement a comprehensive form of model-guided therapy (MGT) that extends beyond the scope of image guidance and can be used as a decision support system in the treatment of patients.

It is our hypothesis that if we can utilize patient modeling techniques to generate valid DPMs (which factor in age, physiologic condition, disease and co-morbidities, genetics, biomarkers and responses to previous treatments), we may be able to develop a statistically valid methodology, on an individual basis, (1) to predict certain diseases or conditions, (2) to predict certain treatment outcomes, (3) to prevent certain diseases or complications and (4) to develop treatment regimens that are personalized for that particular patient. We are calling this proposed system model-based medical evidence (MBME) and are engaged in its development. It is further postulated that the multi-entity Bayesian networks (MEBNs) used in the construction of the DPM will be utilized in the development of a practical decision support system.

The information presented in this review will be used to identify the patient attributes, or information entities (IEs), that will be used to populate the patient databases and MEBNs required for generating DPMs to facilitate data mining and decision support. For any given patient with HCC, the DPM will need to be continuously updated to ensure appropriate guidance of the patient throughout the course of their disease. The development of an ITS-PM for HCC will provide a comprehensive system to identify and then determine the relative value of the wide number of IEs. This system will be designed to provide unified access to general medical and patient-specific information for medical researchers and health care providers from different disciplines including hepatologists, gastroenterologists, medical and surgical oncologists, liver transplant teams, interventional radiologists and radiation oncologists.

The ‘Review’ section of this article is divided into four sections:

1. Review of MGT and the proposed IT framework

2. Review of HCC and its current management

3. Identification of IEs relating to HCC

4. Outlook and expert recommendations for PPPM and HCC

For a more in-depth presentation of the topics discussed in this review article, the reader is referred to reference [3].

### Review of model-guided therapy and the proposed information technology framework

In the approach to MGT taken here, there are three basic structures that will be described. The first is the DPM, which may be regarded as the basic model for representing a patient. The second structure is a therapy imaging and model management system (TIMMS), which provides the hardware and software components for MGT. The third structure to be reviewed is the MEBN, which provides a statistically valid framework for handling medical information.

#### The digital patient model

**Figure 1:**
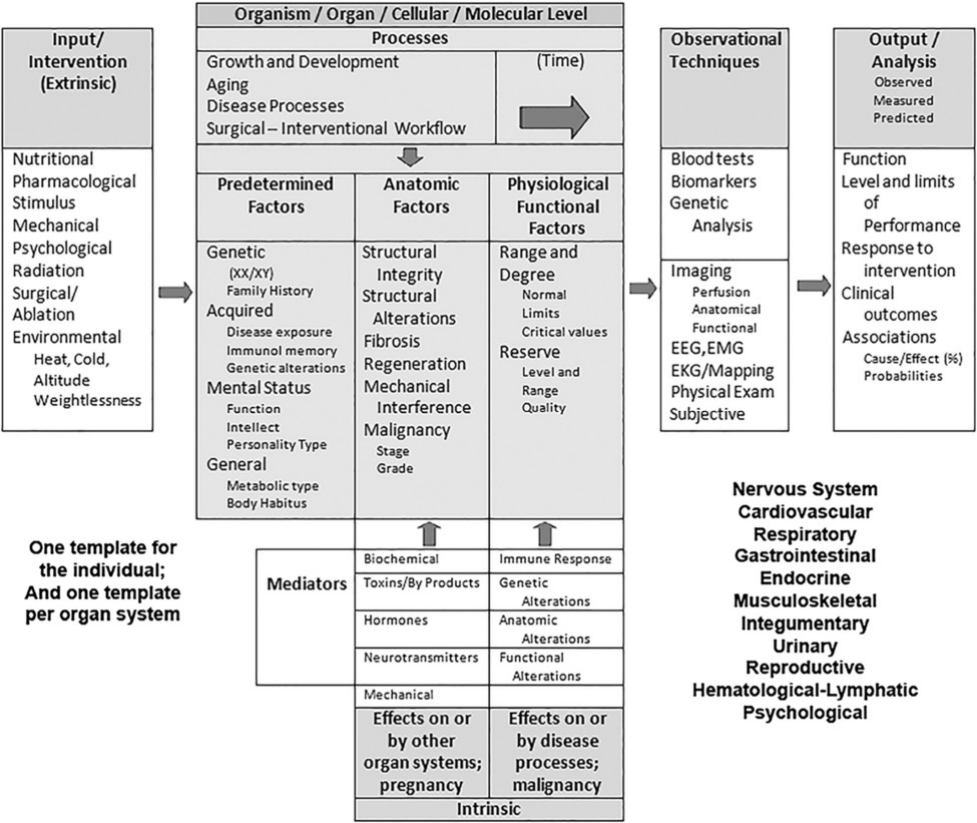
**A generic PSM template.** The DPM is generated when the specific factors and values are entered into the full set of templates (one for each organ system) and the output is analyzed and may be used for prediction.

The DPM provides an IT tool needed for collecting, combining, statistically analyzing, validating and processing patient attributes and biomarkers into a comprehensive, understandable, dynamic, real-time view of the patient. Creation and maintenance of a DPM for any given patient is built upon a generic patient-specific model (PSM) which is modified by the addition of actual patient-specific data, thereby providing a multiscalar, precise, personalized representation of the patient [6,7].A general template for a range of attributes included in a generic PSM is presented in Figure 1. The central area of the template represents the current status of the PSM and is divided into three categories: predetermined factors, anatomic factors and physiologic/functional factors. Allowances are made for those influences that have a direct role in altering the PSM—processes, such as ageing, growth and development, diseases and surgical procedures; extrinsic inputs or interventions; and intrinsic mediators. A separate section of the template displays the PSM output that generates the current or working model of the actual patient or the DPM.

Features of the patient that are amenable to graphical representation are maintained as references within the DPM database structure to picture archiving and communications systems (PACSs) and repositories that allow access to the actual images. This is achieved through functionalities of a suitable IT infrastructure for simulations and interventions (i.e. TIMMS, see below). This allows medical images to be loaded into advanced medical workstations for purposes of treatment planning and simulation, as well as allowing real-time interaction with hardware and software for image-guided interventions, such as radiation therapy and minimally invasive therapies.

Those features of the DPM that are measurable and/or have cause-and-effect relationships, and can be quantified, may be dealt with in a different manner than nonquantifiable features, such as constitution and appearance. In Bayesian terms, these quantifiable features, or information entities (IEs), may be thought of as a dynamic set of data elements, i.e. attributes with interconnected and fluctuating probability distributions. These features will be stored in databases and repositories and will provide the substrate for the MEBN (see below).

#### Therapy imaging and model management system

**Figure 2:**
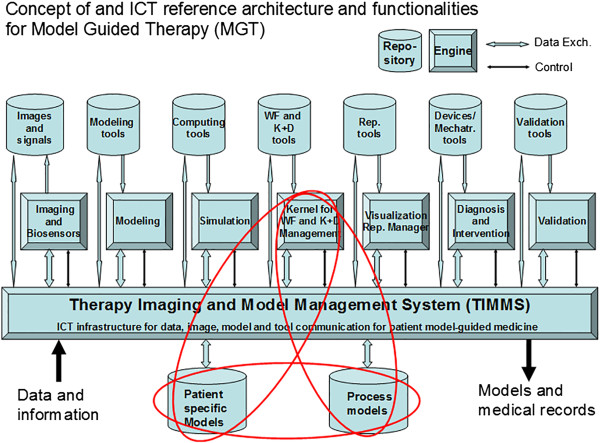
**The structure of the therapy imaging and model management system (TIMMS).** The TIMMS may provide much of the IT framework for personalized medicine. The Kernel for workflow and knowledge and decision management, the patient-specific models repository and the process models repository are the central components for the development of digital patient models through the process of patient-specific modeling. *ICT* information and communication technology; *WF* workflow; *K* + *D* knowledge and decision; *Rep* representation; *Mechatr* mechatronic.

A therapy imaging and model management system (TIMMS) and its application as a surgical assist system for achieving MGT have been described [6,7]. A TIMMS is a comprehensive medical-surgical communication and assist system (Figure 2) that is composed of interconnected computer hardware and software components (such as engines, agents, repositories and IT infrastructure) and provides a wide variety of features and functions throughout the course of medical and surgical treatment. A TIMMS serves as a real-time knowledge management and decision support system, with central functions regulated by the Kernel for workflow and knowledge and decision management, promoting optimized diagnostic, prognostic and therapeutic decisions throughout the treatment workflow.

The Kernel (or ‘brain’ of the system) provides the strategic intelligence for therapeutic planning and workflow execution. The Kernel may use different forms of logic, different database structuring, agents, machine learning and other forms of artificial intelligence, depending on the specific medical application being performed. Agents may be defined as software modules, containing some form of intelligence, which, with some degree of autonomy and adaptability, carry out functions or tasks.

In addition to the Kernel, there are six TIMMS engines that may be defined as software modules that can be executed on an appropriate computing machine to provide functionalities. These engines relate to imaging and biosensor data acquisition, modeling, simulation, workflow and knowledge and decision management, visualization, intervention and validation.

The DPM is maintained by agents regulated by the Kernel in conjunction with the modeling engine and tools, based on data stored in the repositories and/or available from electronic medical records and other sources.

#### Multi-entity Bayesian network

Acceptance, understanding and factoring of the uncertain nature of medical knowledge is a key feature of MGT. The probability distribution of each attribute or variable of the DPM reflects and represents the state of uncertainty associated with the knowledge about a particular feature of an individual patient. The existence of relationships among attributes, represented by appropriate links and their binding strength, are also subject to probability distributions. The value of each attribute probability distribution lies within a statistically definable range of normal and abnormal values. The boundaries of the values for each attribute, and the volatility of the changes of these values, vary in health and disease, and at different ages, and may be subject to further alterations based on the body's homeostatic mechanisms, as well as constitutional, genetic and epigenetic, and environmental factors, including prior medical and surgical interventions. It is therefore reasonable to assume that the described situation surrounding the patient is amenable to be represented by a form of Bayesian network. However, standard Bayesian networks that have been utilized in previous medical decision support and knowledge management systems are inadequate for this purpose. A multi-entity Bayesian network (MEBN) [8] has been developed that can overcome the limitations of standard Bayesian networks.

A MEBN is a logic system that integrates first-order logic with Bayesian probability theory and can provide a descriptive and functional framework for the quantifiable components or IEs of the DPM. These IEs will be stored as attributes within both a PSM database and the appropriate nodes of the graph created for the MEBN. With the addition of sufficient context-appropriate patient-specific data, it is hypothesized that the MEBN will provide a flexible and sufficiently accurate model of a patient and will also provide the necessary framework for the associated situational awareness and decision support that will be required for the performance of MGT.

#### An IT system for predictive, preventive and personalized medicine for HCC

The development of an IT system for predictive, preventive and personalized medicine (ITS-PM) for HCC will provide a comprehensive system to identify and then determine the relative value of the wide number of variables or IEs: (1) factors reflecting clinical assessment of the patient including functional status, liver function, degree of cirrhosis and co-morbidities; (2) factors reflecting tumor biology, at a molecular, genetic and anatomic level; (3) factors reflecting tumor burden and individual patient response; and (4) factors reflecting medical and operative treatments and their outcomes.

The ITS-PM for HCC will provide unified access to general medical and patient-specific information for medical researchers and health care providers from different disciplines including hepatologists, gastroenterologists, medical and surgical oncologists, liver transplant teams, interventional radiologists, and radiation oncologists.

In order for the ITS-PM to handle vast amount of information, we will need to define and develop new types of database solutions and end-user applications. The database solutions should include certain features—easily accessible links to data sources and repositories, functionality that is well organized and easily expandable, the facility for queries that will promote probabilistic and statistically valid investigations and features to facilitate decision support and research.

Beyond the selection and development of database systems, the larger task is to find a way of using IT to pool, integrate and correlate the following: (1) the clinical information relating to diagnosis and treatment of HCC; (2) the research data relating to epidemiology, virology and pathology at the anatomic, molecular and genetic levels; and (3) the role of MGT and patient-specific modeling.

**Figure 3:**
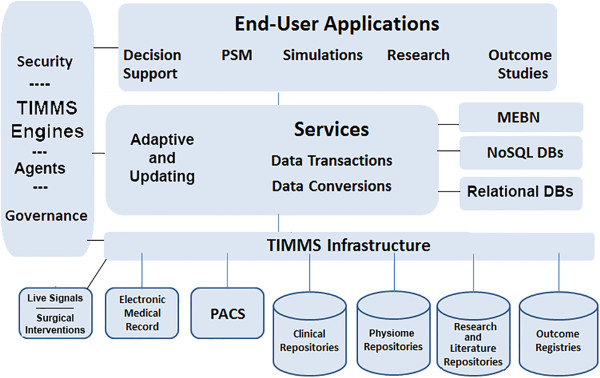
**A schematic for organization of an ITS-PM.** This diagram reorganizes many of the TIMMS components in a structure that will enable the secure interchange of information between data sources, database management systems, data analysis systems and end-user applications. (*PSM* patient-specific model; *TIMMS* therapy and imaging model management system; *PACS* picture archiving and communications system; *MEBN* multi-entity Bayesian network; *NoSQL* not only structured query language; *DBs* databases.

One of our goals is to propose a realistic, plausible approach to the development of DPMs, based on a complex of database and knowledge management systems capable of data storage, data mining, data analysis and decision support. It is imperative that comprehensive and cohesive hardware and software architecture is provided for the ITS-PM to allow each section to function independently, while synchronized and in communication with each other section. At this time, there is no system or collection of systems on the market that can accomplish these tasks. However, Reference Model for Open Distributed Processing (RM-ODP) and service-oriented architecture (SOA) (which may be considered a subset of RM-ODP and is perhaps more widely known) are standards, methodologies or approaches to enterprise system development that could help fulfill the necessary requirements [9,10].It is beyond the scope of this article to provide a complete RM-ODP enterprise proposal with detailed SOA schema. However, at this stage of development, a simplified schematic for the organization of an ITS-PM is presented in Figure 3 for illustrative purposes.

### Review of HCC and its current management

In this section, the clinical information required for constructing a preliminary MEBN for HCC will be reviewed and presented. Appropriate IEs will be extracted from the data presented here, so that in future works, a MEBN and a generic PSM template for HCC can be developed.

Once the preliminary MEBN and PSM template have been established, the addition of patient records, images and outcomes stored in the appropriate TIMMS repositories will allow the creation of a functioning MEBN. It is our goal to utilize this IT approach to medical management to maximize the use of established medical information, to assist decision making, to advance our understanding of health and disease, especially when there is incomplete medical knowledge, and to facilitate PPPM.

#### Hepatocellular carcinoma

HCC is the most common form of primary liver cancer, the fifth most common cancer worldwide and the second leading cause of cancer death in the world [11]. Due to its aggressive nature and poor survival rate, incidence and mortality rates are almost equivalent, accounting for approximately 500,000 deaths annually. Since HCC is most often seen in patients with chronic liver disease or cirrhosis, the incidence of HCC and the epidemiology of underlying liver diseases are closely linked.

The development of HCC is currently felt to take place in a sequential process, with transformation from a small monoclonal dysplastic focus (<1 mm) to a low-grade dysplastic nodule (LGDN) (>1 mm) and then to a high-grade dysplastic nodule (HGDN) before it evolves into a true carcinoma [12]. One of the distinguishing features of early HCC is stromal invasion into the portal tracts. In addition, early diagnosis of HCC is supported by molecular markers (glypican 3, heat shock protein 70 (Hsp70) and glutamine synthetase). If two of these three markers are positive, a sensitivity of 72% and specificity of 100% are reached for the diagnosis of HCC [12]. Transformation into carcinoma is further accompanied by the recruitment of an arterial blood supply, venous invasion and, finally, metastasis. Some of these features will be exploited in the early detection of HCC by contrast-enhanced computed tomography (CT) and magnetic resonance imaging (MRI).

There is evidence that oxidative stress and chronic inflammation form common carcinogenic risk factors in all primary liver cancers. Chronic viral hepatitis B and C, alcoholic and non-alcoholic steatohepatitis, metabolic diseases and mutagens, such as aflatoxins, are the most important risk factors for the development of HCC. A number of genetic markers have been associated with poor prognosis in HCC (more rapid progression, earlier recurrence after surgical resection or transplant) and include primarily keratin 19 (K19), keratin 7 (K7), EpCAM, AE1-AE3, alpha-fetoprotein (AFP), MRP1 and vimentin. In addition, high expression of adenosine triphosphate-binding cassette (ABC) transporters, such as MDR1, ABCG2 and ABCC2, renders the cells resistant to chemotherapy, including cisplatin and doxorubicin, which can be reversed with inhibitors or by using an antisense approach [12].

The relationship between HCC and infection with hepatitis B virus (HBV) and hepatitis C virus (HCV) and with cirrhosis is of paramount importance. Up to 80% of HCC is attributable to HBV or HCV worldwide [13,14]. The risk of HCC is increased 5- to 15-fold in chronic HBV carriers [15,16] and 11.5- to 17-fold in HCV-infected patients [16,17]. Antiviral therapy is effective in preventing HCC in only a small proportion of patients [18,19], and sustained clearance of HBV or HCV may be difficult to accomplish, particularly among cirrhotic patients. Of all HCCs, 80–90% develops in a cirrhotic liver [20]. After 20–30 years of chronic infection, 20–30% of patients develop liver cirrhosis. HCC develops at an annual rate of 1–7% in HCV-infected cirrhotic patients [14] and 3–8% in HBV-infected cirrhotic patients [21].

Screening for HCC in high-risk individuals is recommended since early identification of small tumors has been reported to lead to improved survival [22,23]. High-risk individuals include hepatitis B carriers and those with cirrhosis caused by HCV, alcoholic liver disease, genetic hemochromatosis, primary biliary cirrhosis and autoimmune hepatitis [24]. Ultrasound (US) has been recommended as a non-invasive imaging modality for screening for HCC, with a >60% sensitivity and >90% specificity in detecting HCC [25]. US has been recommended as a non-invasive imaging modality for screening for HCC, with a >60% sensitivity and >90% specificity in detecting HCC [25].

AFP is a serologic marker that is elevated in many patients with HCC and is usually diagnostic in patients with serum levels >500 mcg/L [26]. However, due to its limited sensitivity and specificity, AFP should not be used alone as either a screening agent or diagnostic tool [27]; however, AFP may be helpful in making a diagnosis of HCC in conjunction with imaging modalities.

**Figure 4:**
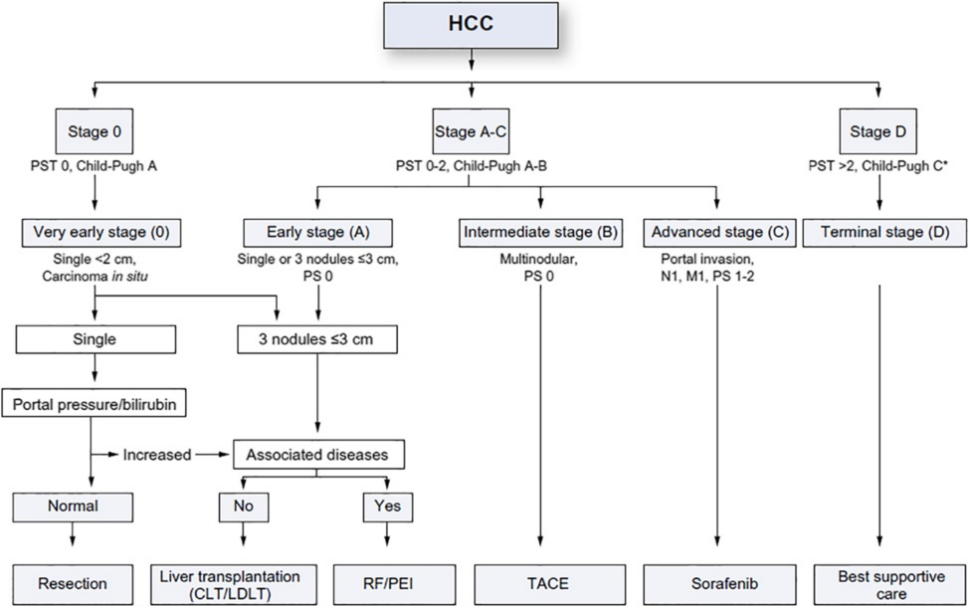
**The Barcelona Clinic Liver Cancer (BCLC) staging system for hepatocellular carcinoma, revised 2011.** *PST* performance status; *CLT/LDLT* cadaver liver transplant/living donor liver transplant; *RF/PEI* radiofrequency ablation/percutaneous ethanol injection; *TACE* transarterial chemoembolization [29].

There are a number of systems available for staging and classifying HCC. We will focus on the Barcelona Clinic Liver Cancer (BCLC) staging system and its updates [28-31] (Figure 4). The BCLC staging system is based on performance status, extent of tumor including presence or absence of vascular invasion, bilirubin level, presence or absence of portal hypertension and Okuda stage. This system is advantageous in that it classifies patients as very early, early, intermediate, advanced or terminal stages thereby establishing a link between stage of disease and appropriate treatment modalities. Very early stage disease is difficult to diagnose since patients are Child-Pugh class A, have no clinical features of liver disease and have a single HCC lesion <2 cm. If diagnosis is made at this stage, the treatment of the tumor has a theoretical 5-year survival rate of 100% [28-31]. Early-stage disease includes patients with up to three nodules, each less than 3 cm, and well-preserved liver function (Child-Pugh class A and B).

Appropriate treatment for early-stage disease includes resection, liver transplantation or ablation with a 5-year survival rate approaching 75% [28-31]. Those with intermediate disease are Child-Pugh class A or B and have large (>5 cm) or multifocal disease without vascular invasion or intrahepatic spread. Transarterial chemoembolization (TACE) is most appropriate for these patients as a means to prolong survival, but not necessarily as a long-term cure [28-31]. Advanced-stage disease includes patients who have cancer-related symptoms, vascular invasion or extrahepatic spread of tumor. Although cure is not a realistic goal in most of these patients, treatment with TACE [32] or sorafenib [33], an oral multikinase inhibitor, may prolong life. Finally, patients who have extensive disease (extensive tumor involvement, Child-Pugh class C) are classified as having terminal stage disease and have a <10% 1-year survival [32]. Treatment goals for these patients should be geared towards palliation and management of symptoms.

#### Radiologic assessment of hepatocellular carcinoma

In the setting of a multidisciplinary clinical liver cancer centre, radiologists play an essential role in the different phases of HCC patients' management, including diagnosis, staging, treatment planning and evaluation of response to treatment.

According to the BCLC staging system, treatment decisions and prognosis are strongly influenced by the tumor extension, in terms of lesions' number and size, presence of macrovascular invasion and extrahepatic tumor spread; precise tumor identification is therefore mandatory for proper patient allocation [28]. Moreover, treatment is often determined by other parameters that are not specifically addressed in the BCLC algorithm, such as tumor location, biliary dilatation, ascites, co-morbidities and radiological response to previous treatments. Therefore, in clinical practice, clinical data need to be fully integrated to an entire spectrum of radiological parameters.

The development of a neoplasm in cirrhosis is a long-lasting process. Many cellular changes occur along the pathway from normal hepatocytes to neoplastic cells so that different types of nodules can be detected in a cirrhotic liver, ranging from regenerative nodules to low-grade dysplastic nodules (LGDNs) and high-grade dysplastic nodules (HGDNs), early HCC and, finally, overt HCC.

HGDNs and early HCC are considered as premalignant and early malignant nodules. Foci of HCC can be found inside HGDNs, while in early HCC, cell degeneration is usually not already associated with the typical vascular changes found in overt HCC [34,35]. These vascular alterations include the reduction of portal venous supply and the development of unpaired arteries and arteriovenous shunts [35]. These typical vascular changes account for the pathological background for current non-invasive diagnosis of HCC at dynamic contrast-enhanced imaging, based on the so-called typical vascular pattern, characterized by wash-in in the arterial phase and wash-out in the portal venous/late phases. This pattern has shown up to 100% specificity for HCC nodules >1 cm in size, in the setting of a cirrhotic liver [36-38].

Only two thirds of HCCs are reported to show a typical vascular pattern, and diagnosis of atypical HCC nodules remains a controversial issue, with differences in their suggested management according to the available guidelines [39]. While both European Association for the Study of the Liver (EASL) and American Association for the Study of Liver Diseases (AASLD) guidelines suggest biopsy for all atypical nodules >1 cm [29-31], the EASL guidelines suggest the use of new diagnostic tools, such as magnetic resonance (MR) using reticuloendothelial system (RES) or hepatocyte-specific contrast agents or contrast-enhanced ultrasound (CEUS) using Sonazoid [40], while biopsy should be performed in case of inconclusive findings.

#### New diagnostic tools

Despite being very specific, the diagnosis of HCC based solely on the detection of neoangiogenesis has a low sensitivity. Thus, the role of different diagnostic elements is under evaluation [36]. In this setting, MR seems to provide some advantages compared to multidetector computed tomography (MDCT), due to its intrinsic capability of identifying other intracellular components, such as glycogen, hemorrhage, water and metals, and defining other parameters, such as diffusivity and biliary function [41-43].

**Diffusion-weighted imaging** Diffusion-weighted imaging (DWI) is a dedicated MR sequence that allows for the evaluation of the random motion (related to thermal effects) of water molecules (‘Brownian motion’) within biological tissues. Recently, DWI has been introduced in liver MR protocols, as several studies have reported its usefulness in improving detection and characterization of focal liver lesions, by measuring their apparent diffusion coefficient [44,45], providing an adjunctive tool in the differential diagnosis between benign and malignant lesions.

**Hepatospecific contrast agents for MR** During carcinogenesis, together with neoangiogenesis, progressive loss of biliary polarization of the hepatocyte and derangement of its microscopic, secretory structure are observed. Recent studies have described modifications of membrane carriers (such as organic anionic transporter protein [OATP] and multidrug-resistance protein [MRP]) that are involved in bilirubin metabolism in neoplastic nodules.

The recent introduction of hepatobiliary contrast agents in MR studies, especially of the highly lipophilic compound Gd-EOB-DTPA, has provided an additional tool for the assessment of the metabolic function of nodules. In fact, due to a competitive binding to bilirubin transporters, these agents provide information regarding the residual performance of cellular membrane proteins and intracellular metabolic activities [46,47].

Moreover, these agents enable the evaluation of both dynamic vascular and metabolic nodular functions in a single-session study since the contrast is taken up within functioning hepatocytes and then excreted at the level of the biliary pole at the end of the intravascular phase. This metabolic phase occurs 20 to 40 min after the injection.

In recent studies, the lack of contrast agent uptake in the hepatobiliary phase has been found in premalignant HGDNs, as well as cases of malignant degeneration (early/overt HCC), even in the absence of the typical vascular pattern [8,36,37,48]. Thus, the use of hepatobiliary contrast agents might increase MR sensitivity in identifying malignant and premalignant lesions. Accordingly, recent studies have demonstrated that the combination of DWI and MR with hepatospecific contrast agents can provide information regarding the risk of premalignant lesions evolving into overt HCC [42,43].

Radiological findings that may be used to generate IEs are summarized in Table 1.

**Table 1:** Radiological features that may be employed as information entities

**Clinical task**	**Radiological findings**
Preprocedural assessment	(a) Number and size of HCC nodules; (b) number and size of nodules considered at risk for neoplastic degeneration; (c) presence and extension of portal vein neoplastic thrombosis; (d) presence of extrahepatic tumor spread; (e) radiological signs of cirrhosis (including varices and ascites); (f) biliary dilatations; (g) radiological signs of co-morbidities
Treatment planning	(a) Features of nodules such as location, degree of vascularization and presence of pseudocapsule; (b) vascular mapping; (c) technical details of previous treatments
Evaluation of previous treatment	(a) Complications; (b) tolerability and compliance; (c) radiological response

#### Role of positron emission tomography

In the last two decades, the development of positron emission tomography (PET) and then PET with computed tomography (PET/CT) imaging has had a large impact on the management of a number of cancer types. PET/CT imaging benefits from the possibility to obtain both structural (CT) and functional (PET) cancer information at the same time. PET obtains images of the biodistribution of radioactive labeled compounds (radiopharmaceuticals) that can be designed to target different biological processes.

Several radiopharmaceuticals are available for PET for imaging various aspects of cancer biology such as cell proliferation and DNA synthesis, tumor hypoxia, tumor angiogenesis and cell apoptosis. However, in current clinical cancer imaging, most PET imaging studies are performed using an analogue of glucose, fluorodeoxyglucose (FDG), labelled with the radioactive Fluorine-18 [^18^ F]. Imaging with FDG is particularly useful because following malignant transformation, various tumors are characterized by increased glucose utilization that is reflected by increased uptake and accumulation of FDG.

In oncology, PET imaging with FDG often provides more sensitive and more specific information about the extent of disease than morphologic/anatomic imaging alone. The metabolic activity of neoplastic tissue measured by PET offers information about cancer biology and aggressiveness and has proven to offer, in comparison to other imaging modalities and for most cancer types, an improved ability to differentiate benign from malignant lesions and therefore to identify early truly neoplastic disease. PET offers also an earlier and often better assessment of response to treatment and an overall better accuracy to restage disease after completion of a treatment course. This in turn results in an overall improved prognostic evaluation during and after treatment.

#### Chemotherapy for HCC

Among the major risk factors for HCC is infection with HBV or HCV. These particular hepatic pathogens are involved in a multifaceted process that involves a number of alterations that are genetic and epigenetic, such as the activation of cellular oncogenes and the inactivation of tumor suppressor genes.

Systemic therapy with single-agent or combination chemotherapy has been studied extensively. However, cytotoxic chemotherapy traditionally has been associated with low response rates and questionable disease control. Although systemic chemotherapy has a limited role in HCC, recent advances in molecular targeted therapies appear to be leading to an increasing role for chemotherapy.

New data on the efficacy of molecularly targeted agents have brought these agents, particularly sorafenib, to the forefront of therapy for advanced HCC. Sorafenib is a multitargeted, orally active, small molecule tyrosine kinase inhibitor (TKI) that inhibits Raf kinase and the VEGFR intracellular kinase pathway [49].

Sorafenib offers the potential for prolonged survival, although objective tumor remissions are scarce. This molecular targeted therapy would be the first-line treatment in patients with Child-Pugh B cirrhosis and advanced HCC who are not candidates to undergo liver transplant, surgical resection, TACE or radiofrequency ablation (RFA). Most physicians would not prescribe sorafenib for patients with HCC and Child-Pugh C cirrhosis, due to the significantly abnormal liver function and the high risk of treatment-related toxicity.

Systemic chemotherapy is an option for patients whose tumors progress while on sorafenib and whose performance status and liver function are sufficient to tolerate it. The best regimen is not established. The side effect profile of each individual regimen must be carefully considered in patients who have advanced liver disease and/or a short life expectancy. Doxorubicin monotherapy is rarely used in the treatment of HCC. Given the cardiotoxic side effects and low response rates, the treatment would be considered for patients who failed molecular targeted therapy and still exhibit a moderate/fair performance status, with fairly intact liver and cardiac function. 5FU/leucovorin treatment candidates would include the elderly, poor performance status patients and those who are classified as Child-Pugh class C.

#### Surgical treatment of hepatocellular carcinoma

The treatment of HCC has undergone evolution and refinement over the past three decades. Changes in the understanding of HCC in the context of a wide variety of factors such as tumor size, number and location, underlying liver function and portal pressure and hepatic anatomy, in combination with refinement of surgical techniques and technologies, have greatly influenced the approach to surgical management.

Surgery is considered the mainstay of curative HCC treatment with resection and transplantation achieving the best outcomes in well-selected candidates (5-year survival of 60–80%) [29]. In general, surgical resection of HCC, especially within the Milano/Mazzaferro criteria for liver transplantation (i.e. solitary tumor ≤5 cm or up to three tumors all ≤3 cm) in patients with well-preserved underlying liver function (Child-Pugh A and selectively B patients), offers the greatest chances for survival, while liver transplantation, in patients with compromised liver function (Child-Pugh B/C), is generally considered the treatment of choice. It is important to note that these recommendations are undergoing constant reassessment and revision. The application of specific techniques, such as RFA and the practice of reclassification of patients with well-compensated liver function, have, in some reports, suggested alternative treatment protocols.

The following areas have been identified as key issues relating to PPPM and surgical treatment for HCC:

1. Tumor characterization, such as size, number and vascular invasion

2. The patient's clinical status, particularly the presence of cirrhosis, the degree of portal hypertension and liver functional reserve

3. Preoperative management, such as patient selection for resection or transplantation, choice of donor, down-staging and bridging therapies

4. Surgical techniques, including techniques to minimize blood loss during surgery and to ensure an adequate liver remnant

### Ablation therapies for hepatocellular cancer

A wide variety of minimally invasive, or locoregional, therapies are now available for the treatment of HCC along with surgery, systemic chemotherapy and, occasionally, radiation therapy. These therapies include thermal ablation, including radiofrequency ablation (RFA), interstitial laser thermotherapy (ILT), microwave ablation (MWA), high-intensity focused ultrasound (HIFU) and cryotherapy (CRYO) and nonthermal ablations including alcohol injection (ETOH), irreversible electroporation (IRE) and photodynamic therapy (PDT).

At this time, RFA has the largest clinical experience and will serve as the prototype for understanding the principles, mechanisms and methods that have been developed for the treatment of HCC. The effectiveness of RFA, and of more recent forms of ablation therapy, will be reviewed. Percutaneous, catheter-directed therapy, or transarterial chemoembolization (TACE), and catheter-directed radiation therapy with Yttrium-90 will be reviewed below.

**Introduction to tumor ablation** Tumor ablation involves a focal destruction of tissue to achieve a therapeutic effect. The targeted tissue is focal as well, demonstrable under direct visualization, palpation or via radiologic imaging; multiple foci are individually targeted. The therapeutic effect may be an attempt at local cure of a tumor or may be for debulking a tumor for symptomatic (i.e. pain) reasons. The term ablation suggests immediacy to the effect, while the results of a tumor ablation may ‘mature’ post-procedurally; the primary direct effects are usually most notable.

Ablation can be practiced in various venues: by a surgeon in the operating room, in an open procedure or under laparoscopy or by an interventional radiologist in a modern hybrid suite or in a simple procedure room. For our purposes here, we discuss image-guided thermal ablation. This suggests an interventional setting in which US, CT, fluoroscopy, MRI or PET could be the imaging modality of choice. In such a setting, the ablation is performed with a minimally invasive approach—the effect is delivered interstitially and intratumorally via a device placed percutaneously. In a percutaneous procedure, imaging plays a critical role in the targeting of the lesion, the protection of the surrounding anatomy and, if possible, in the monitoring and control of the ablation process.

**Forms of ablation** Thermal ablations take advantage of a number of energy sources including electric current (RFA), electromagnetic radiation (MWA, LASER) and mechanical waves (HIFU). Tissue death occurs at 60°C, but in clinical use, the probes generate over 90°C temperatures. Contemporary cryotherapy uses a closed circuit of high-pressure gas to draw heat from tissue. Cell death takes place at temperatures below -40°C. These various thermal agents have each been engineered into various delivery devices. Clinical systems to treat tumors in all parts of the body include the liver, kidney, lung, adrenal, bone, soft tissue and more.

The efficacy of RFA is related to the size of the liver tumor. It is accepted that the lesion should not exceed 2.5–3.0 cm to obtain complete necrosis [49]. It has been reported that certain microwave ablation (MWA) devices may allow successful treatment of lesions as large as 5 cm with an acceptable margin of safety [50]. Notwithstanding, RFA commonly is utilized for lesions greater than 3 cm in diameter, occasionally for palliative debulking rather than for cure.

Guidelines may assist in the selection and use of the more widely used thermal technologies: RFA, MWA and CRYO. To achieve maximal effectiveness for cure, basic treatment precepts analogous to those in surgery must be understood and adhered to, including the following: (1) proper patient selection, (2) treatment of the entire lesion and (3) providing adequate tumor margins. For ablation therapies, certain additional considerations include (1) avoidance of anatomic structures that influence effective deposition of energy, such as blood vessels that divert energy from the tumor (‘heat sink’ or ‘cold sink’); (2) avoiding tissue alterations that could influence energy deposition, such as too rapid increase in tissue impedance, tissue charring and creation of microscopic gas bubbles; (3) ensuring complete coverage when overlapping zones of treatment are required so that no gaps of inadequately treated tumor cells remain; and (4) ensuring an adequate treatment margin when the tumor is in proximity to sensitive or vital structures (e.g. GI tract or myocardium).

### Catheter-directed delivery of therapy for hepatocellular carcinoma

Techniques have been developed for catheter-directed delivery of therapy for HCC since the 1980s and are still undergoing evolution. This has included bland embolization with particles, as well as delivery of chemotherapeutic agents with a variety of materials, referred to as TACE.

TACE is made both feasible and effective due to the dual blood supply of the liver. HCC derives 95% of its blood supply from the hepatic artery, whereas normal hepatic parenchyma is supplied 75 and 25% by the portal vein and hepatic artery, respectively. (These differences in arterial supply account for the detectability of early HCC on dynamic, contrast-enhanced CT and MRI.) Advances in catheter and guide wire technology have been accompanied by the development of techniques for the superselective placement of catheters for the safe and effective delivery of therapeutic agents to hepatic tumors.

Current recommendations based on the Barcelona Clinic Liver Cancer (BCLC) staging classification and treatment schedule [29-31] advise TACE for patients with intermediate stage (Okuda stage 1–2; Childs-Pugh stage A to B; performance status 0) with multinodular HCC. Childs-Pugh Class B and C cirrhotic patients, as well as patients with end-stage HCC, are at an increased risk for liver failure and death and are not appropriate candidates for TACE [24].

Combination therapy with RFA and TACE may lead to more extensive tumor necrosis than mono-ablative therapy and may be a more effective treatment for HCC [51]. For patients with single nodules between 3 to 5 cm in size, the combination of RFA and TACE has proven to be more effective compared to RFA alone [52,53]. The intermediate stage is composed of a very heterogeneous population, ranging from patients with a single large nodule to patients with multifocal extensive bilobar involvement. In clinical practice, there is wide variation in the management of these patients. Single large nodules can be treated effectively by surgical resection, down-staging followed by liver transplant (in highly selected patients) or by combining TACE and RFA [54]. Further study will be needed to determine the effectiveness of combining RFA and TACE and in which order.

The combination of TACE with antiangiogenic agents, such as sorafenib, is under investigation as well [55,56]. The use of sorafenib may curtail the post-TACE rise in VEGF-mediated signaling and simultaneously target tumor foci distant from the site of treatment.

### Radiation therapy for hepatocellular carcinoma

Optimizing the therapeutic ratio and achieving an safety profile in the treatment of HCC with radiation therapy (RT) has historically been a challenge. Although HCC is a radiosensitive tumor, it is surrounded by highly radiosensitive organs, including the remainder of the liver and hollow gastrointestinal viscera. As technology has advanced to the point of allowing a highly conformal dose to be delivered to the tumor while sparing the surrounding normal tissues, radiotherapy has re-emerged as a viable treatment modality for many patients with HCC, pending randomized controlled trials to confirm its efficacy.

#### Stereotactic body radiotherapy

Stereotactic body radiotherapy (SBRT), which involves the precise delivery of highly conformal, image-guided, ablative doses of external beam radiation in an abbreviated course of five fractions or less, has been shown to be an effective alternative to other ablative procedures in nonsurgical candidates with tumors up to 6 cm in size. To ensure that enough residual liver is spared from RT, it is important to keep the target volume as small as possible, which has been made feasible through the use of advanced treatment-planning technologies like multiphasic and multimodality imaging, breathing motion management, highly conformal plans and image-guided treatment delivery. The safety and efficacy of SBRT has been shown in several prospective studies of metastatic lesions in noncirrhotic livers [57,58], and in the past few years, data has also emerged from several groups reporting success in treating HCC with SBRT in patients with cirrhosis.

Child-Pugh class is an important predictor of morbidity for patients undergoing SBRT, and while there is sufficient safety data in class A, SBRT should be used with caution (or not at all) in classes B and C. The presence of portal vein thrombosis does not impact the safety or efficacy of SBRT. Other procedural considerations to prevent toxicity, which are not contraindications per se, include keeping an interval of 14 days between SBRT and chemotherapy and 6 months between SBRT and any local embolization procedure. There may also be some situations in which SBRT is technically feasible, but systemic therapy or best supportive care is more appropriate than any local therapy, including patients with life expectancy <12 weeks, or patients with progressive or untreated gross extrahepatic disease.

**Radioembolization** The technique of radioembolization is similar in many ways to any other embolization procedure (e.g. TACE), in that it involves catheter-based infusion of particles targeted at the branches of the hepatic artery that feed the portion of the liver where the tumor is located. However, the mechanism of action in radioembolization is primarily due to radiation-induced necrosis and does not rely on ischemia secondary to reduced blood flow to achieve tumor necrosis. There are two different radioisotopes used for radioembolization worldwide, iodine-131[I-131]-labeled Lipiodol and yttrium-90[Y90]-labeled microspheres. Since radioembolization has minimal embolic effect, and will not obstruct the blood supply to the functional liver, it is often considered the safer alternative to TACE for tumors with portal vein thrombosis.

Prior to treating a patient, it is important to perform a 99^m^-Tc macroaggregated albumin (MAA) scan to demonstrate that there is no shunting of blood flow to the lung or gastrointestinal tract that cannot be corrected by catheter techniques. The potential for ≥30 Gy radiation exposure to the lung is considered an absolute contraindication to radioembolization. Relative contraindications include a limited hepatic reserve, irreversibly elevated bilirubin, and prior RT involving the liver.

According to the Radioembolization Brachytherapy Oncology Consortium Consensus Guidelines, radioembolization may be used in patients with unresectable primary HCC with liver-dominant tumor burden and life expectancy >3 months [59].

### Identification of information entities relating to hepatocellular cancer

**Figure 5:**
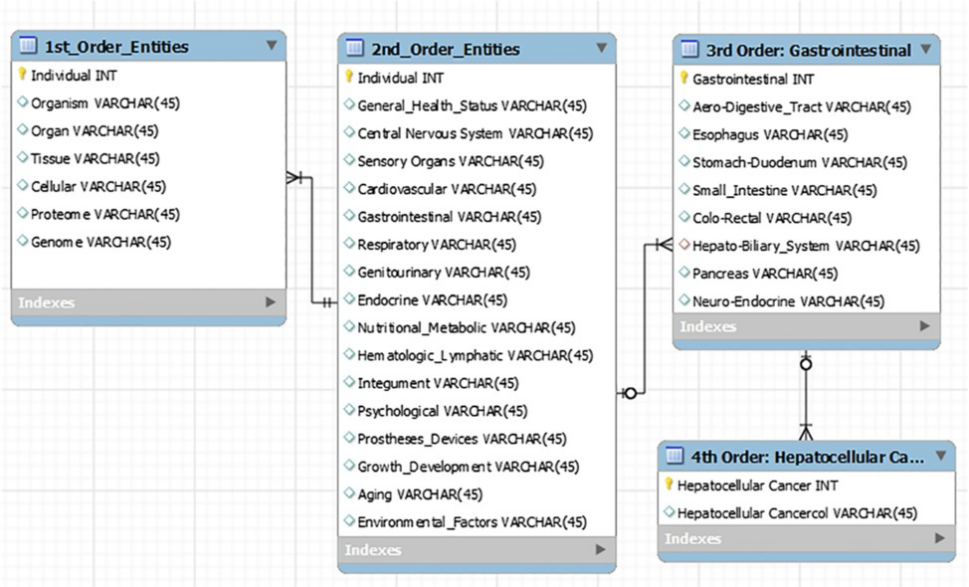
**A portion of a simplified entity-relationship diagram for a relational database is shown displaying the 1st-, 2nd- and 3rd-order information entities of a generic patient-specific model and a 4th-order entity: hepatocellular carcinoma.** *INT* integer; *VARCHAR* includes text [characters, numbers and punctuation].

**Figure 6:**
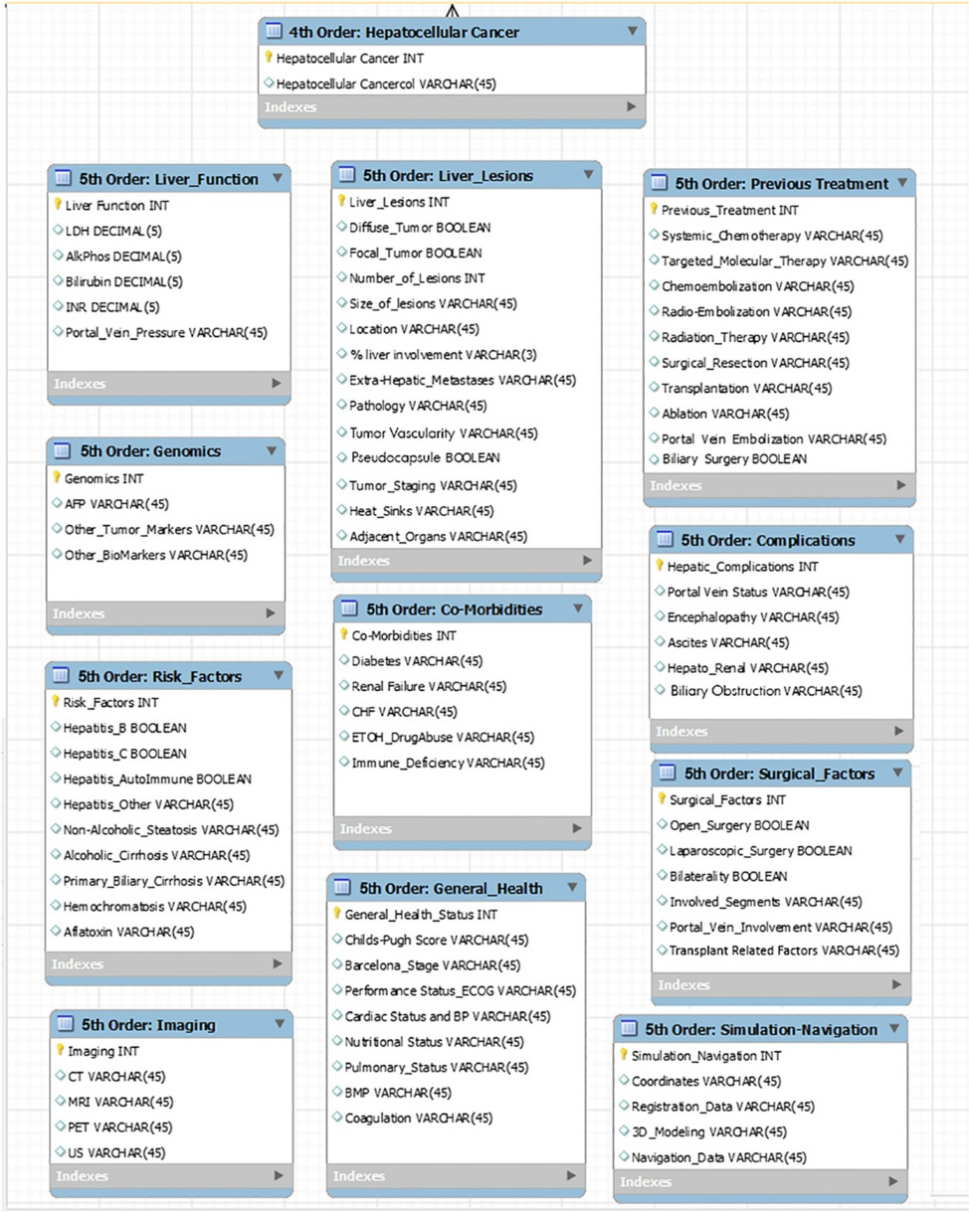
A portion of a simplified entity-relationship diagram for a relational database is shown displaying the 5th-order information entities relating to hepatocellular carcinoma.

**Figure 7:**
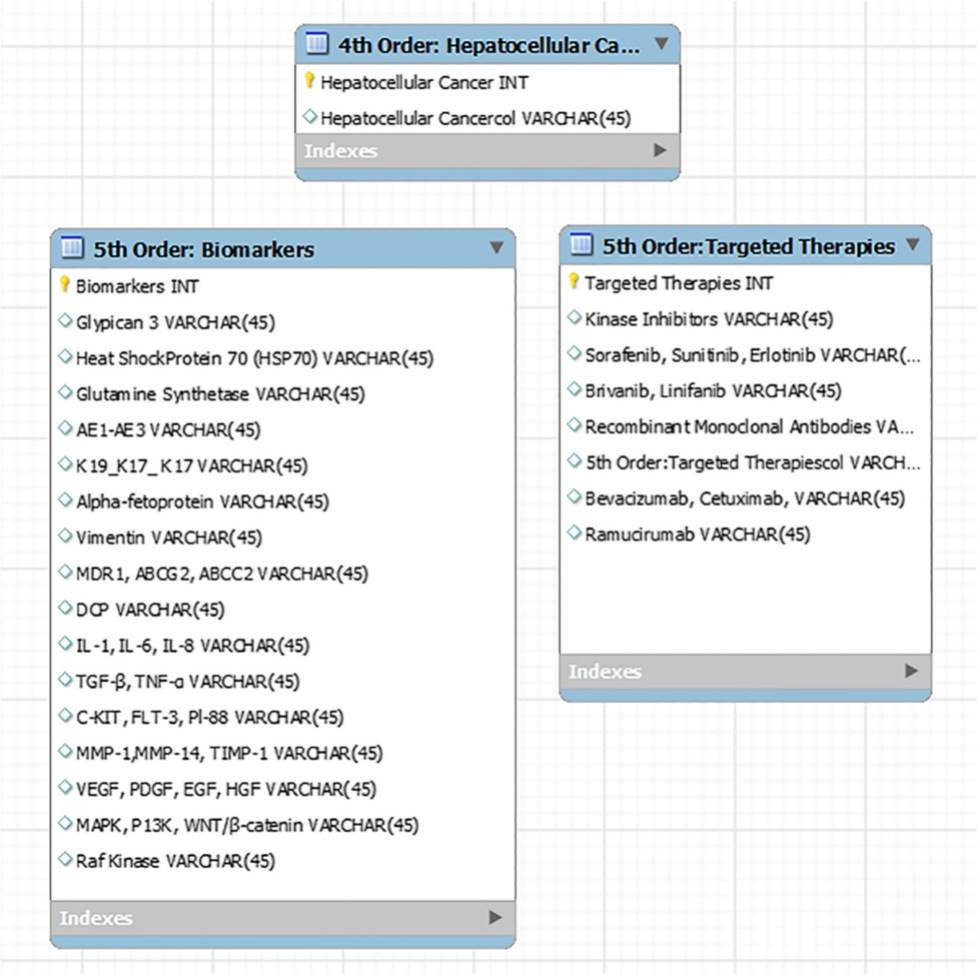
A portion of a simplified entity-relationship diagram for a relational database that may be linked to a graph database for research in biomarker and targeted therapies is shown displaying the 5th-order information entities relating to biomarkers and targeted therapies for HCC.

In the preceding sections, we have defined the current state of knowledge, as well as the limits of our knowledge, with respect to HCC. To advance PPPM, we will be examining HCC from a more integrated point of view, combining epidemiology, risk factors, infectious etiologies, pathology, microenvironment and biomarkers, screening and diagnostic technologies and treatment modalities (single, combined and/or sequential). The IEs identified will be used in organizing and populating the databases required for the development of the DPMs and MEBNs for an ITS-PM, as described above.First- and second-order IEs for the generic PSM for HCC are identified in the first two columns in Figure 5. The attributes of these entities may be obtained through links with the appropriate databases, spreadsheets, electronic medical records (EMRs) and repositories, for example, by means of the functionalities of a TIMMS. These first- and second-order IEs are broken down into third-order IEs and further subdivisions that include the vast, and continually expanding, list of patient-related information.Fourth- and fifth-order IEs for HCC, as identified in ‘Review of HCC and its current management’ section of this article, are identified in Figure 6. The attributes of these entities may also be determined through links with the appropriate databases, spreadsheets, electronic medical records (EMRs) and repositories by means of TIMMS functionalities.Figure 7 identifies fourth- and fifth-order IEs for HCC relating to potential biomarkers and targeted therapies. It is postulated that a comprehensive DPM incorporating the combined IEs will provide insights facilitating and enhancing diagnosis and treatment of HCC, as well as providing a means of handling research data relating to epidemiology, virology and pathology at the anatomic, molecular and genetic levels.

As these IEs are fully identified, the next goal will be the development of the databases that incorporate these patient-specific IEs and the development of MEBNs, DPMs and an ITS-PM. It is important to keep in mind that a MEBN is a logic system that integrates first-order logic with Bayesian probability theory [8] and can provide a descriptive and functional framework for the DPMs. It is the nature of a MEBN to increase in overall accuracy, according to Pearl's Bi-directional Belief Updating Algorithm [60,61], as more experience and evidence are added to the system and the precision of the data used to generate the various component probabilities increases. As the databases are populated with data from a growing number of patient records, and as more DPMs are developed, it will then become possible to begin the study and validation of PPPM through MGT. We have called this approach model-based medical evidence (MBME). With the addition of sufficient context-appropriate patient-specific data, it is hypothesized that the combined components of the ITS-PM will provide a flexible and sufficiently accurate model of a patient and will also provide the necessary framework for the associated situational awareness and decision support that will be required for the performance of PPPM. The development of MBME will be augmented by the incorporation of information extracted from RCTs, as well as other data sources. The ultimate goal of MBME is to promote an approach to patient care based on a personalized, as well as global, understanding of diseases and of treatment outcomes, in a way that broadens the scope of evidence-based medicine. It may be possible to retrospectively study large groups of patients who have received certain treatments and identify subpopulations of patients who have responded favorably. If the population of patients is sufficiently large, evaluation of the DPMs of these patients may yield constellations of IEs that may provide prognostic information for future patients.

### Outlook and expert recommendations for PPPM and HCC

In this concluding section, the proposed benefits of the ITS-PM will be presented in the form of outlook and expert recommendations for PPPM and HCC. It has been our assumption that the development of a comprehensive IT system will allow the accumulation and organization of vast amounts of patient-specific information and will be designed to perform statistically valid data synthesis and processing. It is intended that this system helps lead to more personalized patient evaluation and treatment choices rather than patient management based primarily upon local availability and expertise. It is hypothesized that through the accumulation of sufficiently large numbers of DPMs, constellations of IEs will be identified that provide prognostic information that is patient-specific.

In the short term, more immediate benefits of developing an ITS-PM for HCC may include enhancements in screening, use of targeted therapies, combined locoregional therapies and flexible and expanded treatment algorithms.

#### Extensions to the BCLC staging system algorithm

**Figure 8:**
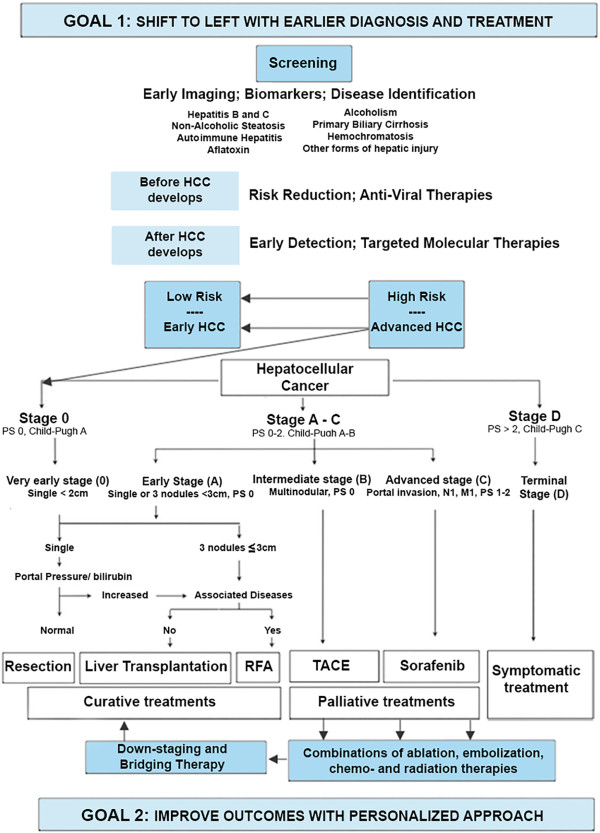
**It may be possible to build on the BCLC staging system platform with an IT system for personalized medicine (ITS-PM).** The first goal will be, through enhanced screening, to have patients seek medical attention earlier in the course of their disease, so that they may enter the algorithm at a more favorable stage; i.e. a ‘shift to the left’. The second goal will be to improve outcomes through a better understanding of treatment subcategories, combined treatments and the effects of down-staging.

The BCLC staging system algorithm is an important advancement in the application of the principles of PPPM in cancer treatment. The IEs identified in ‘Identification of information entities relating to hepatocellular cancer’ section may be used to expand and reinforce the algorithm in several ways. Extensions may be added to the algorithm (Figure 8) that (1) facilitate enhanced screening for HCC, (2) explore ways in which targeted therapies may be used to improve outcomes and (3) provide statistically validated evidence regarding the selection of the best treatment from the many options available for palliative, down-staging and bridging therapies.

**Enhanced screening and shift to the left** It may be possible to positively influence where in the BCLC staging system a patient enters the algorithm. It would be desirable to achieve a shift to the left with a greater number of patients seeking medical attention at a stage of the disease when there is a greater chance of cure. As more data are accumulated, it may become possible to evaluate current screening techniques and criteria, and to improve them in light of accumulated information, with the establishment of more comprehensive programs for earlier and more effective screening.

**Possible expanded role of targeted therapies** Currently, targeted therapies for HCC are finding applications in patients with advanced disease. However, it may also be possible to expand the role of targeted therapies in the future to influence the development or progression of disease in earlier stages. It may even become possible to shift high-risk patients into low-risk categories, thereby averting the development of HCC altogether.

**Improved outcomes with further personalized approach** The BCLC staging system algorithm is concerned primarily with the initial treatment choices for patients with HCC. While several treatment options are indicated for patients with intermediate and advanced disease, the complexities relating to these treatment options have not yet been developed within the context of the algorithm (although these issues are discussed elsewhere [29-31]). The ITS-PM may be employed to generate MBME to obtain a better understanding of the role of locoregional treatment options and combined treatments and the effectiveness of down-staging and/or bridging therapies to achieve improved outcomes. The algorithm may be expanded to include, in greater detail, the currently available treatments for HCC, individually or in combination (Figure 8).

#### New algorithms

Patients with HCC are treated according to the best available practices with the intention of achieving cure and/or improved quality and length of life. Additional algorithms are presented here that are based on current best practices. These algorithms relate to alternative and/or combined therapies for patients with early, intermediate and advanced stages of diseases. They may be used for palliative treatments, for down-staging and for selecting treatment options with patients with progression of disease. Treatments for HCC included in these algorithms include surgical resection, liver transplantation, percutaneous ablation, transarterial chemoembolization (TACE), local radiotherapy with yttrium-90 microspheres, stereotactic body radiation therapy (SBRT) and systemic targeted therapy with the oral multikinase inhibitor, sorafenib.

Selection of the best regimen of therapy for patients with intermediate disease is unclear at this time, due to insufficient available information. However, it is felt that as more patients are entered into the ITS-PHC, and as detailed patient-specific models can be developed in sufficient numbers, it will be possible to assess and validate the treatment choices and criteria depicted in these algorithms in a manner that heretofore has not been achievable. Target benchmarks for the effective treatment of hepatocellular carcinoma can be established. Comparative studies of the costs of different treatment protocols may be evaluated with respect to successes and failures in treatment outcomes and with respect to overall quality of care and the patient's quality of life.

**Figure 9:**
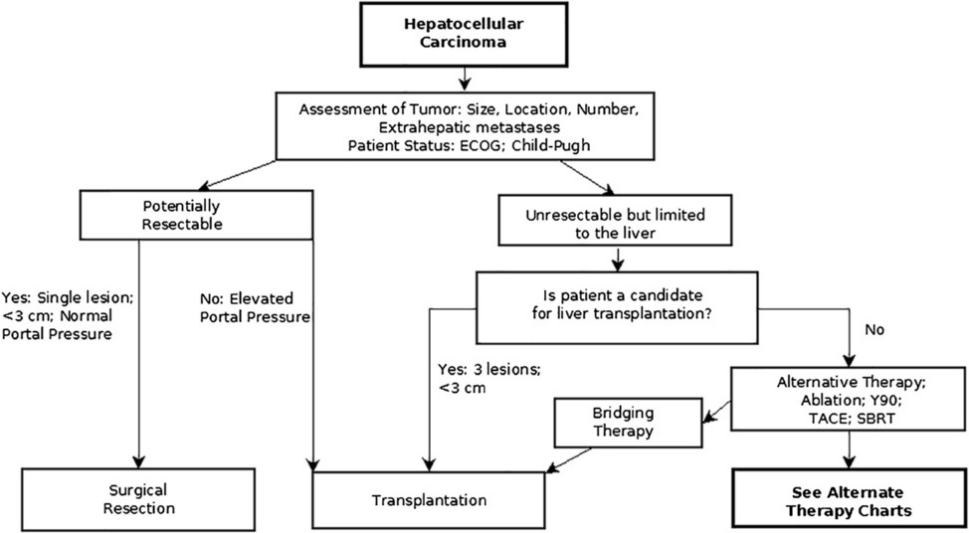
**A wider and more flexible assortment of alternative therapies and bridging therapies are introduced in this algorithm.** This algorithm continues in Figures 10 and 11 (alternative therapy charts: single and multiple lesions).

**Figure 10:**
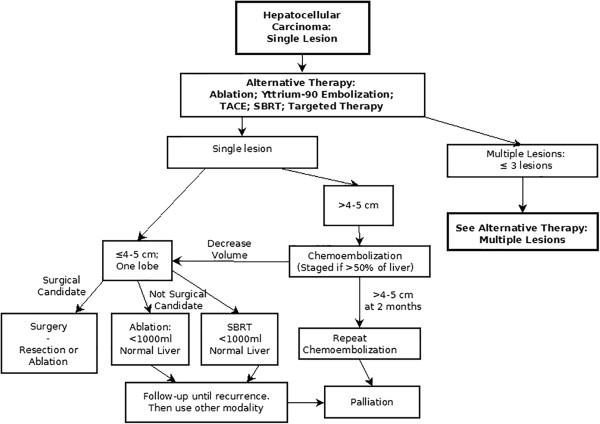
Alternative therapy chart: single lesions.

**Figure 11:**
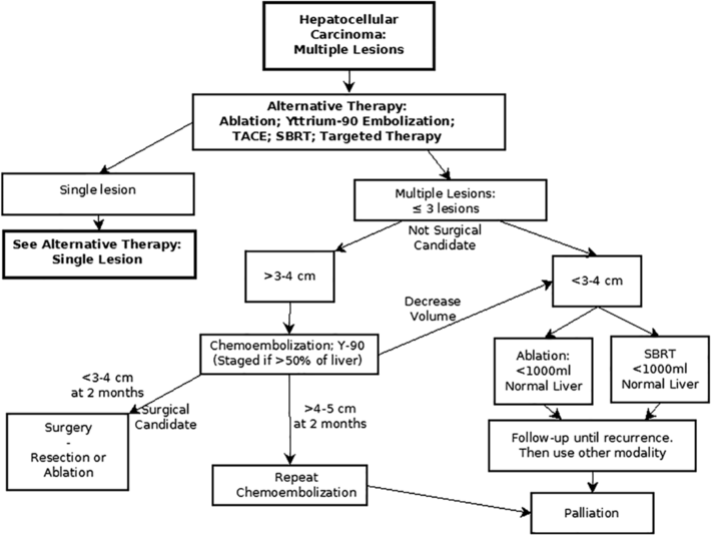
Alternative therapy chart: multiple lesions.

New algorithms are presented here that address issues and patient selection relating to alternatives in palliative treatments and efforts to achieve down-staging. Currently available treatments for HCC included in these algorithms are surgical resection, liver transplantation, percutaneous ablation, TACE, radioembolization with yttrium-90 microspheres, stereotactic body radiation therapy (SBRT) and systemic targeted therapy with the oral multikinase inhibitor, sorafenib. In accordance with the BCLC staging system, the efficacy and safety of each treatment modality depends on the stage of liver disease, performance status of the patient and severity of underlying liver disease.Treatment algorithms are presented here (Figures 9, 10 and 11) that represent extensions to the BCLC staging system algorithm by providing a flexible approach to alternative therapies and by introducing the possibility of down-staging and bridging therapy.

These algorithms present treatment pathways based on the current literature and provide a framework for the accumulation of data regarding specific treatment protocols. The outcomes of these varied treatments can be linked to extensive data collected for each patient (according to the categories itemized in Figures 5, 6 and 7) within the ITS-PM.

## Conclusions

It is the goal of personalized medicine to identify the best diagnostic tests and treatment modalities objectively, given the patient's actual clinical status. We have presented an approach to advancing the care of patients with HCC, building on the well-established BCLC staging system, by proposing extensions to include enhanced screening and a means to evaluate more specific treatment regimens. This approach will require the development of a comprehensive ITS-PM that will utilize a computing system for patient modeling and decision support.

The ITS-PM system presented here is in its earliest stages of development, as is the DPM, which is the basic unit for this system. It is the goal of this project to develop a system of Bayesian inference built on large databases of individual patients that will provide a way of giving greater emphasis to individual patient characteristics in expanded patient screening programs and treatment algorithms.

If sufficient data regarding each patient are collected, it may be possible to generate knowledge that may be generalized to achieving a more complete understanding of the pathophysiology, prevention and treatment of HCC. This may be accomplished through the use of enhanced IT with modeling and inference techniques, thus creating a new methodology for evidence-based medicine utilizing model theory, i.e. model-based medical evidence.

In addition, as more information is gathered and validated through the ITS-PM, target benchmarks for the effective treatment of hepatocellular carcinoma can be established. Comparative studies of the costs of different treatment protocols may be evaluated with respect to successes and failures in treatment outcomes and with respect to overall quality of care and the patient's quality of life.

The tasks that lay before us in the immediate future include the development of the databases that incorporate patient-specific IEs and the development of the MEBN and the associated decision support. As these are populated with data from a growing number of patient records, DPMs will be developed. It will then become possible to begin the study and validation of predictive, preventive and personalized medicine through model-guided therapy and model-based medical evidence.

## Abbreviations

AASLD: American Association for the Study of Liver Diseases; AFP: Alpha-fetoprotein; BCLC: Barcelona Clinic Liver Cancer; CEUS: Contrast-enhanced ultrasound; CRYO: Cryotherapy; CT: Computed tomography; DPM: Digital patient model; DWI: Diffusion-weighted imaging; EASL: European Association for the Study of the Liver; EMR: Electronic medical records; EPMA: European Association for Predictive, Preventive and Personalised Medicine; ETOH: Alcohol injection; FDG: Fluorodeoxyglucose; HBV: Hepatitis B virus; HCC: Hepatocellular carcinoma; HCV: Hepatitis C virus; HGDN: High-grade dysplastic nodule; HIFU: High-intensity focused ultrasound; IE: Information entity; ILT: Interstitial laser thermotherapy; IRE: Irreversible electroporation; IT: Information technology; ITS-PM: Information technology system for predictive, preventive and personalized medicine; LGDN: Low-grade dysplastic nodule; MAA: Macroaggregated albumin; MBME: Model-based medical evidence; MDCT: Multidetector computed tomography; MEBN: Multi-entity Bayesian networks; MGT: Model-guided therapy; MRI: Magnetic resonance imaging; MWA: Microwave ablation; PSM: Patient-specific model; PACS: Picture archiving and communications systems; PDT: Photodynamic therapy; PET: Positron emission tomography; PET/CT: PET with computed tomography; PPPM: Predictive, preventive and personalized medicine; RCT: randomized controlled trial; RFA: Radiofrequency ablation; RM-ODP: Reference Model for Open Distributed Processing; SBRT: Stereotactic body radiotherapy; SOA: Service-oriented architecture; TACE: Transarterial chemoembolization; TIMMS: Therapy imaging and model management system; US: Ultrasound; Y90: Yttrium-90.

## Competing interests

The authors declare that they have no competing interests.

## Authors’ contributions

LB contributed to the sections on the digital patient model, model-guided therapy, patient assessment, ablation therapy, catheter-guided therapies, surgical treatment, IT system and expert recommendations. HUL contributed to the sections on the digital patient model and model-guided therapy, IT system and expert recommendations. SM contributed to the sections on hepatocellular carcinoma and patient assessment, surgical treatment, catheter directed therapies and expert recommendations. SV contributed to the sections on hepatocellular carcinoma and patient assessment. DD and HH contributed to the sections on hepatocellular carcinoma and patient assessment and personalized chemotherapy for hepatocellular carcinoma. IB, VB, DC and CB contributed to the sections on radiologic assessment of hepatocellular carcinoma. GE contributed to the section on PET/CT imaging in hepatocellular carcinoma. SS contributed to the section on surgical treatment for hepatocellular carcinoma. PM and EvS contributed to the sections on ablation therapies for hepatocellular carcinoma. MDM contributed to the section on radiation oncology in the treatment of hepatocellular carcinoma. HA contributed to the sections on radiation oncology in the treatment of hepatocellular carcinoma and expert recommendations. All authors read and approved the final manuscript, with the exception of PM who passed away prior to the completion of this article.

## References

[B1] Golubnitschaja O Costigliola V EPMA General report & recommendations in predictive, preventive and personalised medicine 2012: white paper of the European Association for Predictive, Preventive and Personalised Medicine EPMA J 2012 3 14 10.1186/1878-5085-3-14 23116135 PMC3485619

[B2] Berliner L Lemke HU van Sonnenberg E Ashamalla H Mattes MD Dosik D Hazin H Shah S Mohanty S Verma S Esposito G Bargellini I Information and communication technology in personalised medicine: a clinical use-case for hepatocellular cancer EPMA J 2014 5 Suppl 1 A59 10.1186/1878-5085-5-S1-A59 PMC4274760 25538797

[B3] Berliner L Lemke HU van Sonnenberg E Ashamalla H Mattes MD Dosik D Hazin H Shah S Mohanty S Verma S Esposito G Bargellini I An Information Technology Framework for PPPM: a Use-Case with Hepatocellular Carcinoma, Advances in PPPM book series 2014 Springer In press

[B4] Armijo D McDonnell C Werner K Electronic health record usability: evaluation and use case framework 2009 Agency for Healthcare Research and Quality (AHRQ), U.S. Department of Health and Human Services Accessed 7/27/2014. ( http://www.himss.org/files/HIMSSorg/content/files/Code%20119%20-%20EHR%20Usability_Evaluation%20and%20Use%20Case%20Framework_AHRQ.pdf)

[B5] Roff JT Module 1. UML fundamentals UML: a Beginner's Guide 2003 Berkley, CA: McGraw-Hill/Osborne

[B6] Lemke HU Berliner L Niederlag W, Lemke HU O, Rienhoff O Personalised medicine and model-guided therapy Personalisierte Medizin und Informationstechnologie, 15 2010 Dresden: Health Academy 39 48

[B7] Lemke HU Berliner L Niederlag W, Lemke HU, Golubnitschaja O, Rienhoff O Personalised medicine and patient-specific modelling Personalisierte Medizin, 14 2010 Dresden: Health Academy 155 164

[B8] Laskey KB MEBN: a language for first-order Bayesian knowledge bases Artif Intell 2008 172 140 178 10.1016/j.artint.2007.09.006

[B9] Linington PF Milosevic Z Tanaka A Vallecillo A Building Enterprise Systems with ODP: an Introduction to Open Distributed Processing 2012 Boca Raton: CRC Press

[B10] Nickul D Reitman L Ward J Wilber J Service Oriented Architecture (SOA) and Specialized Messaging Patterns Technical; White Paper 2007 Adobe Systems Inc http://www.adobe.com/enterprise/pdfs/Services_Oriented_Architecture_from_Adobe.pdf Accessed online 6 January 2009

[B11] Jemal A Bray F Center MM Ferlay J Ward E Forman D Global cancer statistics CA Cancer J Clin 2011 61 69 90 10.3322/caac.20107 21296855

[B12] Roskams T Anatomic pathology of hepatocellular carcinoma: impact on prognosis and response to therapy Clin Liver Dis 2011 15 245 259 10.1016/j.cld.2011.03.004 21689611

[B13] Tsai W-L Chung RT Viral hepatocarcinogenesis Oncogene 2010 29 2309 2324 10.1038/onc.2010.36 20228847 PMC3148694

[B14] Perz JF Armstrong GL Farrington LA Hutin YJ Bell BP The contributions of hepatitis B virus and hepatitis C virus infections to cirrhosis and primary liver cancer worldwide J Hepatol 2006 45 529 538 10.1016/j.jhep.2006.05.013 16879891

[B15] El-Serag HB Rudolph KL Hepatocellular carcinoma: epidemiology and molecular carcinogenesis Gastroenterology 2007 132 2557 2576 10.1053/j.gastro.2007.04.061 17570226

[B16] Donato F Boffetta P Puoti M A meta-analysis of epidemiological studies on the combined effect of hepatitis B and C virus infections in causing hepatocellular carcinoma Int J Cancer 1998 75 347 354 10.1002/(SICI)1097-0215(19980130)75:3<347::AID-IJC4>3.0.CO;2-2 9455792

[B17] Donato F Tagger A Gelatti U Parrinello G Boffetta P Albertini A Decarli A Trevisi P Ribero ML Martelli C Porru S Nardi G Alcohol and hepatocellular carcinoma: the effect of lifetime intake and hepatitis virus infections in men and women Am J Epidemiol 2002 155 323 331 10.1093/aje/155.4.323 11836196

[B18] Sorrell MF Belongia EA Costa J Gareen IF Grem JL Inadomi JM Kern ER McHugh JA Petersen GM Rein MF Strader DB Trotter HT National Institutes of Health consensus development conference statement: management of hepatitis B Hepatology 2009 49 5 Suppl S4 S12 19399804 10.1002/hep.22946

[B19] Tai AW Chung RT Treatment failure in hepatitis C: mechanisms of non-response J Hepatol 2009 50 412 420 10.1016/j.jhep.2008.11.010 19070928 PMC2743341

[B20] Caldwell S Park SH The epidemiology of hepatocellular cancer: from the perspectives of public health problem to tumor biology J Gastroenterol 2009 44 Suppl XIX 96 101 19148801 10.1007/s00535-008-2258-6

[B21] Fattovich G Natural history and prognosis of hepatitis B Sem Liver Diseas 2003 23 47 58 10.1055/s-2003-37590 12616450

[B22] Wong LL Limm WM Severino R Wong LM Improved survival with screening for hepatocellular carcinoma Liver Transpl 2000 6 320 325 10.1053/lv.2000.4875 10827233

[B23] Zhang BH Yang BH Tang ZY Randomized controlled trial of screening for hepatocellular carcinoma J Cancer Res Clin Oncol 2004 130 417 422 15042359 10.1007/s00432-004-0552-0 PMC12161851

[B24] Aronsohn A Mohanty SR Current treatment strategies for hepatocellular carcinoma Curr Cancer Ther Rev 2010 6 199 206 10.2174/157339410791698151

[B25] Bolondi L Sofia S Siringo S Gaiani S Casali A Zironi G Piscaglia F Gramantieri L Zanetti M Sherman M Surveillance programme of cirrhotic patients for early diagnosis and treatment of hepatocellular carcinoma: a cost effectiveness analysis Gut 2001 48 251 259 10.1136/gut.48.2.251 11156649 PMC1728189

[B26] Wu JT Serum alpha-fetoprotein and its lectin reactivity in liver diseases: a review Ann Clin Lab Sci 1990 20 98 105 1691611

[B27] Sherman M Alphafetoprotein: an obituary J Hepatol 2001 34 603 605 10.1016/S0168-8278(01)00025-3 11394662

[B28] Llovet JM Bru C Bruix J Prognosis of hepatocellular carcinoma: the BCLC staging classification Semin Liver Dis 1999 19 329 338 10.1055/s-2007-1007122 10518312

[B29] European Association for the Study of the Liver European Organisation for Research and Treatment of Cancer: EASL–EORTC clinical practice guidelines: management of hepatocellular carcinoma J Hepatol 2012 56 908 943 22424438 10.1016/j.jhep.2011.12.001

[B30] Sherman M Bruix J AASLD PRACTICE GUIDELINE. Management of hepatocellular carcinoma: an update Hepatology 2011 53 1020 1022 10.1002/hep.24199 21374666 PMC3084991

[B31] Bruix J Sherman M AASLD Practice Guideline. Management of hepatocellular carcinoma Hepatology 2010 http://www.aasld.org/practiceguidelines/Documents/Bookmarked%20Practice%20Guidelines/HCCUpdate2010.pdf. Accessed 20 November 2011

[B32] El-Serag HB Marrero JA Rudolph L Reddy KR Diagnosis and treatment of hepatocellular carcinoma Gastroenterology 2008 134 1752 1763 10.1053/j.gastro.2008.02.090 18471552

[B33] Llovet JM Ricci S Mazzaferro V Hilgard P Gane E Blanc JF de Oliveira AC Santoro A Raoul JL Forner A Schwartz M Porta C Zeuzem S Bolondi L Greten TF Galle PR Seitz JF Borbath I Häussinger D Giannaris T Shan M Moscovici M Voliotis D Bruix J SHARP Investigators Study Group Sorafenib in advanced hepatocellular carcinoma N Engl J Med 2008 359 378 390 10.1056/NEJMoa0708857 18650514

[B34] Theise ND Park YN Kojiro M Dysplastic nodules and hepatocarcinogenesis Clin Liver Dis 2002 6 497 512 10.1016/S1089-3261(02)00006-5 12122867

[B35] International Consensus Group for Hepatocellular Neoplasia Pathologic diagnosis of early hepatocellular carcinoma: a report of the International Consensus Group for Hepatocellular Neoplasia Hepatology 2009 49 658 664 19177576 10.1002/hep.22709

[B36] Bartolozzi C Battaglia V Bargellini I Bozzi E Campani D Pollina LE Filipponi F Contrast-enhanced magnetic resonance imaging of 102 nodules in cirrhosis: correlation with histological findings on explanted livers Abdom Imaging 2013 38 290 296 10.1007/s00261-012-9952-9 23053453

[B37] Forner A Vilana R Ayuso C Bianchi L Solé M Ayuso JR Boix L Sala M Varela M Llovet JM Brú C Bruix J Diagnosis of hepatic nodules 20 mm or smaller in cirrhosis: prospective validation of the noninvasive diagnostic criteria for hepatocellular carcinoma Hepatology 2008 47 97 104 18069697 10.1002/hep.21966

[B38] Sangiovanni A Manini MA Iavarone M Romeo R Forzenigo LV Fraquelli M Massironi S Della Corte C Ronchi G Rumi MG Biondetti P Colombo M The diagnostic and economic impact of contrast imaging techniques in the diagnosis of small hepatocellular carcinoma in cirrhosis Gut 2010 59 638 644 10.1136/gut.2009.187286 19951909

[B39] Bolondi L Gaiani S Celli N Golfieri R Grigioni WF Leoni S Venturi AM Piscaglia F Characterization of small nodules in cirrhosis by assessment of vascularity: the problem of hypovascular hepatocellular carcinoma Hepatology 2005 42 27 34 15954118 10.1002/hep.20728

[B40] Omata M Lesmana LA Tateishi R Chen PJ Lin SM Yoshida H Kudo M Lee JM Choi BI Poon RT Shiina S Cheng AL Jia JD Obi S Han KH Jafri W Chow P Lim SG Chawla YK Budihusodo U Gani RA Lesmana CR Putranto TA Liaw YF Sarin SK Asian Pacific Association for the Study of the Liver consensus recommendations on hepatocellular carcinoma Hepatol Int 2010 4 439 474 10.1007/s12072-010-9165-7 20827404 PMC2900561

[B41] Park MJ Kim YK Lee MH Lee JH Validation of diagnostic criteria using gadoxetic acid-enhanced and diffusion-weighted MR imaging for small hepatocellular carcinoma (≤2.0 cm) in patients with hepatitis-induced liver cirrhosis Acta Radiol 2013 54 127 136 10.1258/ar.2012.120262 23148300

[B42] Kim YK Lee WJ Park MJ Kim SH Rhim H Choi D Hypovascular hypointense nodules on hepatobiliary phase gadoxetic acid-enhanced MR images in patients with cirrhosis: potential of DW imaging in predicting progression to hypervascular HCC Radiology 2012 265 104 114 10.1148/radiol.12112649 22891358

[B43] Park MJ Kim YK Lee MW Lee WJ Kim YS Kim SH Choi D Rhim H Small hepatocellular carcinomas: improved sensitivity by combining gadoxetic acid-enhanced and diffusion-weighted MR imaging patterns Radiology 2012 264 761 770 10.1148/radiol.12112517 22843769

[B44] Ichikawa T Cederle MP Grazioli L Marsh W Fibrolamellar hepatocellular carcinoma: pre- and posttherapy evaluation with CT and MR imaging Radiology 2000 217 145 151 10.1148/radiology.217.1.r00se46145 11012437

[B45] Gourtsoyianni S Papanikolaou N Yarmenitis S Maris T Karantanas A Gourtsoyiannis N Respiratory gated diffusion-weighted imaging of the liver: value of apparent diffusion coefficient measurements in the differentiation between most commonly encountered benign and malignant focal liver lesions Eur Radiol 2008 18 486 492 10.1007/s00330-007-0798-4 17994317

[B46] Narita M Hatano E Arizono S Miyagawa-Hayashino A Isoda H Kitamura K Taura K Yasuchika K Nitta T Ikai I Uemoto S Expression of OATP1B3 determines uptake of Gd-EOBDTPA in hepatocellular carcinoma J Gastroenterol 2009 44 793 798 10.1007/s00535-009-0056-4 19404564

[B47] Kitao A Zen Y Matsui O Gabata T Kobayashi S Koda W Kozaka K Yoneda N Yamashita T Kaneko S Nakanuma Y Hepatocellular carcinoma: signal intensity at gadoxetic acid-enhanced MR imaging—correlation with molecular transporters and histopathologic features Radiology 2010 256 817 826 10.1148/radiol.10092214 20663969

[B48] Kim SH Kim SH Lee J Kim MJ Jeon YH Park Y Choi D Lee WJ Lim HK Gadoxetic-acid enhanced MRI versus triple-phase MDCT for the preoperative detection of hepatocellular carcinoma AJR Am J Roentgenol 2009 192 1675 1681 10.2214/AJR.08.1262 19457834

[B49] Liu L Cao Y Chen C Zhang X McNabola A Wilkie D Wilhelm S Lynch M Carter C Sorafenib blocks the RAF/MEK/ERK pathway, inhibits tumor angiogenesis, and induces tumor cell apoptosis in hepatocellular carcinoma model PLC/PRF/5 Cancer Res 2006 66 11851 11858 10.1158/0008-5472.CAN-06-1377 17178882

[B50] Livraghi T Meloni F Solbiati L Zanus G for the Collaborative Italian Group using AMICA system Complications of microwave ablation for liver tumors: results of a multicenter study Cardiovasc Intervent Radiol 2012 35 868 874 10.1007/s00270-011-0241-8 21833809

[B51] Bloomston M Binitie O Fraiji E Murr M Zervos E Goldin S Kudryk B Zwiebel B Black T Fargher S Rosemurgy AS Transcatheter arterial chemoembolization with or without radiofrequency ablation in the management of patients with advanced hepatic malignancy Am Surg 2002 68 827 831 12356160

[B52] Lencioni R Crocetti L Petruzzi P Vignali C Bozzi E Della Pina C Bargellini I Cioni D Oliveri F De Simone P Bartolozzi C Brunetto M Filipponi F Doxorubicin-eluting bead-enhanced radiofrequency ablation of hepatocellular carcinoma: a pilot clinical study J Hepatol 2008 49 217 222 10.1016/j.jhep.2008.03.021 18486261

[B53] Peng ZW Zhang YJ Chen MS Xu L Liang HH Lin XJ Guo RP Zhang YQ Lau WY Radiofrequency ablation with or without transcatheter arterial chemoembolization in the treatment of hepatocellular carcinoma: a prospective randomized trial J Clin Oncol 2013 31 426 432 10.1200/JCO.2012.42.9936 23269991

[B54] Bargellini I Florio F Golfieri R Grosso M Lauretti DL Cioni R Trends in utilization of transarterial treatments for hepatocellular carcinoma: results of a survey by the Italian Society of Interventional Radiology Cardiovasc Intervent Radiol 2014 37 438 444 [Epub ahead of print 2013 May 30] 10.1007/s00270-013-0656-5 23719667

[B55] Lencioni R Management of hepatocellular carcinoma with transarterial chemoembolization in the era of systemic targeted therapy Crit Rev Oncol Hematol 2012 83 216 224 10.1016/j.critrevonc.2011.10.008 22142656

[B56] Lencioni R Llovet JM Han G Tak W-Y Yang J Leberre M-A Niu W Nicholson K Meinhardt G Bruix J Sorafenib or placebo in combination with transarterial chemoembolization (TACE) with doxorubicin-eluting beads (DEBDOX) for intermediate-stage hepatocellular carcinoma (HCC): phase II, randomized, double-blind SPACE trial. [abstract LBA154] J Clin Oncol 2012 30 Suppl 4 40

[B57] Rusthoven KE Kavanagh BD Cardenes H Stieber VW Burri SH Feigenberg SJ Chidel MA Pugh TJ Franklin W Kane M Gaspar LE Schefter TE Multi-institutional phase I/II trial of stereotactic body radiation therapy for liver metastases J Clin Oncol 2009 27 1572 1578 Epub 2009 Mar 2 10.1200/JCO.2008.19.6329 19255321

[B58] Herfarth KK Debus J Lohr F Bahner ML Rhein B Fritz P Höss A Schlegel W Wannenmacher MF Stereotactic single-dose radiation therapy of liver tumors: results of a phase I/II trial J Clin Oncol 2001 19 164 170 11134209 10.1200/JCO.2001.19.1.164

[B59] Kennedy A Nag S Salem R Murthy R McEwan AJ Nutting C Benson A 3rd Espat J Bilbao JI Sharma RA Thomas JP Coldwell D Recommendations for radioembolization of hepatic malignancies using yttrium-90 microsphere brachytherapy: a consensus panel report from the radioembolization brachytherapy oncology consortium Int J Radiat Oncol Biol Phys 2007 68 13 23 10.1016/j.ijrobp.2006.11.060 17448867

[B60] Pearl J Probabilistic Reasoning in Intelligent Systems: Networks of Plausible Inference 1988 San Mateo, CA: Morgan Kaufmann Publishers

[B61] Bayesian networks http://www.pr-owl.org/basics/bn.php#pearl1988 Accessed November 17, 2013

